# Rapid Detection of New Delhi Metallo-β-Lactamase Gene Using Recombinase-Aided Amplification Directly on Clinical Samples From Children

**DOI:** 10.3389/fmicb.2021.691289

**Published:** 2021-07-22

**Authors:** Yanling Feng, Guanhua Xue, Junxia Feng, Chao Yan, Jinghua Cui, Lin Gan, Rui Zhang, Hanqin Zhao, Wenjian Xu, Nannan Li, Shiyu Liu, Shuheng Du, Weiwei Zhang, Hailan Yao, Jun Tai, Lijuan Ma, Ting Zhang, Dong Qu, Yongxiang Wei, Jing Yuan

**Affiliations:** ^1^Capital Institute of Pediatrics, Beijing, China; ^2^Children’s Hospital Affiliated to Capital Institute of Pediatrics, Beijing, China

**Keywords:** carbapenemase, *bla*_NDM_, recombinase-aided amplification, pediatrics, character

## Abstract

New Delhi metallo-β-lactamase, a metallo-β-lactamase carbapenemase type, mediates resistance to most β-lactam antibiotics including penicillins, cephalosporins, and carbapenems. Therefore, it is important to detect *bla*_NDM_ genes in children’s clinical samples as quickly as possible and analyze their characteristics. Here, a recombinase-aided amplification (RAA) assay, which operates in a single one-step reaction tube at 39°C in 5−15 min, was established to target *bla*_NDM_ genes in children’s clinical samples. The analytical sensitivity of the RAA assay was 20 copies, and the various bacterial types without *bla*_NDM_ genes did not amplify. This method was used to detect *bla*_NDM_ genes in 112 children’s stool samples, 10 of which were tested positive by both RAA and standard PCR. To further investigate the characteristics of carbapenem-resistant bacteria carrying *bla*_NDM_ in children, 15 carbapenem-resistant bacteria (*Escherichia coli*, *Klebsiella pneumoniae*, *Acinetobacter baumannii*, *Citrobacter freundii*, *Klebsiella oxytoca*, *Acinetobacter junii*, and *Proteus mirabilis*) were isolated from the 10 samples. Notably, more than one bacterial type was isolated from three samples. Most of these isolates were resistant to cephalosporins, cefoperazone-sulbactam, piperacillin-tazobactam, ticarcillin-clavulanic acid, aztreonam, co-trimoxazole, and carbapenems. *bla*_NDM__–__1_ and *bla*_NDM__–__5_ were the two main types in these samples. These data show that the RAA assay has potential to be a sensitive and rapid *bla*_NDM_ gene screening test for clinical samples. The common existence of *bla*_NDM_ and multi-drug resistance genes presents major challenges for pediatric treatment.

## Introduction

Since their first use in 1985, broad-spectrum carbapenems have been commonly used in clinical practice (Lyon, 1985), but carbapenemase-producing *Enterobacterales* bacteria now pose an increasingly important threat to global health (Trecarichi and Tumbarello, 2017). Carbapenemases are β-lactamases belonging to three different Ambler classes (classes A, B, and D). Class A enzymes include the most common *Klebsiella pneumoniae* carbapenemase (KPC) family and the much less commonly encountered non-metallo-carbapenemase A (NmcA) enzyme, *Serratia marcescens* enzymes (SMEs), the imipenem-hydrolyzing β-lactamase (IMI), and the Guiana extended spectrum (GES) enzyme family. Class B enzymes are metallo-β-lactamases (MBL) and include New Delhi metallo-β-lactamases (NDM), imipenemase (IMP), and Verona integron-encoded metallo-β-lactamase (VIM). Class D enzymes are OXA-type carbapenemases (Tooke et al., 2019).

NDMs can hydrolyze most β-lactams including penicillins, cephalosporins, and carbapenems (Kumarasamy et al., 2010; Khan et al., 2017). The first clinical bacterial strain carrying this gene was isolated in 2009 from a Swedish patient who had traveled to India in 2007, where he acquired a *K. pneumoniae*-related urinary tract infection. Carbapenem resistance in this isolate was mediated through the production of a novel carbapenemase designated NDM-1 (Yong et al., 2009). Since then, NDM-1 has been found in various *Enterobacteriaceae*, and in *Acinetobacter* and *Pseudomonas* species, and 31 NDM variants have thus far been identified (Hong et al., 2015; Farhat and Khan, 2020).

NDM-positive bacteria, which include *Enterobacteriaceae*, *Pseudomonas*, and *Acinetobacter*, cause various infections with high mortality rates (Rahman et al., 2018; Yang et al., 2020). Therefore, the rapid and sensitive detection of these NDM-positive pathogens is required if appropriate therapy is to be administered. Recombinase-aided amplification (RAA) is a new assay based on isothermal amplification technology. The reaction system includes a recombinase (UvsX), a single-stranded DNA-binding protein, and a DNA polymerase. The amplification process is completed within 15–30 min at 39°C, making it suitable for clinical applications (Zhang et al., 2017; Fan et al., 2019; Qi et al., 2019; Shen et al., 2019; Wang et al., 2019; Xue et al., 2020a,b).

In the present work, we developed an RAA assay to detect the *bla*_NDM_ gene in clinical samples. The analytical specificity and sensitivity of the assay were evaluated, and the detection results were PCR verified. To further analyze the characteristics of carbapenem-resistant strains with *bla*_NDM_ from children, the minimum inhibitory concentrations (MICs), *bla*_NDM_ genotypes and clinical diagnosis pertaining to these isolates were investigated.

## Materials and Methods

### Bacteria

Altogether, 16 common *Enterobacterales* bacterial types (*Klebsiella pneumoniae* 2146, *Acinetobacter baumannii* 1 and 2, *Pseudomonas aeruginosa*, *K. pneumoniae* 700603, *Escherichia coli* 25922, *Klebsiella oxytoca*, *P. aeruginosa* ATCC27853, *Shigella sonnei*, *Salmonella enteritidis*, *Enterobacter aerogenes*, *Proteus mirabilis*, *Campylobacter jejuni*, *Enterobacter cloacae*, *S. marcescens*, *Citrobacter freundii*) were investigated in this study, the sources of which are listed in Table 1. These bacteria were cultured at 37°C in brain heart infusion medium (BHI, Oxoid Ltd., United Kingdom) broth. From them, *K. pneumoniae* 2146, *A. baumannii* 1, and *P. aeruginosa* contain *bla*_NDM__–__1_ genes, *A. baumannii* 2 contains *bla*_*KPC*__–__2_ gene, whereas the other bacterial types were all sensitive strains. All bacterial types included in this study were previously evaluated for the presence of *bla*_NDM_ by PCR (data not shown).

**TABLE 1:** Bacterial types used in this study.

**Species**	**Source**
*Klebsiella pneumoniae* 2146	Our microorganism center
*Acinetobacter baumannii* 1	Clinical isolate
*Pseudomonas aeruginosa*	Clinical isolate
*Escherichia coli* ATCC 25922	Our microorganism center
*K. pneumoniae* ATCC 700603	Our microorganism center
*Acinetobacter baumannii* 2	Our microorganism center
*Pseudomonas aeruginosa* ATCC 27853	Our microorganism center
*Shigella sonnei*	Our microorganism center
*Salmonella enteritidis*	Our microorganism center
*Klebsiella oxytoca*	Clinical isolate
*Enterobacter aerogenes*	Clinical isolate
*Proteus mirabilis*	Clinical isolate
*Enterobacter cloacae*	Clinical isolate
*Serratia marcescens*	Clinical isolate
*Campylobacter jejuni*	Clinical isolate
*Citrobacter freundii*	Clinical isolate

### Clinical Samples and Bacterial Cultures

One hundred and twelve stool samples were randomly collected from each inpatients in the Capital Institute of Pediatrics, Beijing, China. All specimens were first cultured on blood plates and the isolated bacteria were verified using the VITEK 2 compact system (BioMerieux, France). The rest were used for DNA isolation. A 200-mg sample was taken from each remaining fecal sample for DNA extraction, and the instructions from the kit (Tiangen Biotech Co., Ltd., Beijing, China) were followed.

### Primer Design

The sequences of all 31 *bla*_NDM_ genes were downloaded from the National Center for Biotechnology Information (NCBI) GenBank database.^1^ The conserved regions in these 31 genes were used to manually design the primers and the probe according to the principles relating to RAA primer and probe design (primer size between 30 and 35 bp, probe size between 46 and 52 bp, product size between 100 and 200 bp). The conserved region in the 16S rRNA gene was designed to act as an internal positive control. An NCBI primer-specific BLAST analysis was used to confirm the specificity of the primers and probe. Online OligoEvaluator software^2^ was used to analyze the potential for primer dimers to occur and to identify hairpins. All primers and probes were synthesized and purified by Sangon Biotech (Shanghai, China) using high-performance liquid chromatography.

### DNA Extraction

Total DNA was extracted from each bacterial type (Table 1) with the QIAamp DNA Mini Kit (Qiagen, Hilden, Germany) in accordance with the manufacturer’s instructions. The DNA samples were eluted in 150 μL of nuclease-free water and stored at −80°C until use. *Klebsiella pneumoniae* 2146 DNA was 10-fold diluted from 10^–7^ copies/μL to 10^0^ copies/μL, as calculated using the following formula: DNA copy number (copy number/μL) = [6.02 × 10^23^ × plasmid concentration (ng/μL) × 10^–9^]/[DNA length (in nucleotides) × 660], and then stored at −80°C until use.

### Recombinant Plasmid Construction

The full-length *bla*_NDM__–__1_ gene (*K. pneumoniae* 2146, GenBank Accession No. CP006659) was PCR-amplified and cloned into vector pUC57 (Tiangen Biotech Co., Ltd., Beijing, China). The standard recombinant plasmids with 10-fold concentrations ranging from 10^7^ copies/μL to 10^0^ copies/μL were prepared and stored at −80°C until use.

### RAA Assay

RAA assays were performed in 50-μL reaction volumes using a commercial RAA kit (Jiangsu Qitian Bio-Tech Co., Ltd., China). The reaction mixtures contained 2 μL of extracted DNA template, 25 μL of reaction buffer, 15.7 μL of DNase-free water, 2.1 μL of primer F (10 μM), 2.1 μL of primer R (10 μM), 0.6 μL of the probe (10 μM), and 2.5 μL of 280 mM magnesium acetate. The reaction mixture was added to a tube containing the RAA enzyme mix in a lyophilized form. Tubes were placed into a B6100 Oscillation mixer (QT-RAA-B6100, Jiangsu Qitian Bio-Tech Co., Ltd., China) and incubated for 4 min, mixed briefly, centrifuged, and finally transferred to a fluorescence detector (QT-RAA-1620, Jiangsu Qitian Bio-Tech Co., Ltd.) to be measured for 20 min at 39°C.

### Analytical Sensitivity and Specificity of the RAA Assay

The analytical sensitivity of the RAA assay was determined using 10-fold serial dilutions of the recombinant plasmid ranging from 10^7^ copies/μL to 10^0^ copies/μL. Assay specificity was evaluated by testing the bacterial panel described in Table 1. *K. pneumoniae* 2146 and two other *bla*_NDM__–__1_-containing clinical isolates (*A. baumannii* 1 and *P. aeruginosa*) were used as the positive controls for testing the specificity of the RAA assay toward *bla*_NDM_. Distilled water was used as the negative control.

### PCR Detection and Sequencing

A 25-μL reaction volume containing the following components was used for all the PCRs: 12.5 μL of PCR Master Mix reagent (Tiangen Biotech Co., Ltd., Beijing, China), 9.5 μL of double-distilled water, 0.5 μL of 10 μM NDM-F primer (5′-ATGGAATTGCCCAATATTAT-3′) and NDM-R primer (5′-TCAGCGCAGCTTGTCGGCCA-3′), and 2 μL of DNA template. The PCR cycling conditions were 94°C for 2 min, followed by 35 cycles at 94°C for 30 s, 55°C for 30 s, and 72°C for 45 s. The final extension step was 72°C for 10 min. PCR products were electrophoretically separated on 1.5% agarose gels and stained with ethidium bromide. Images were documented on the Gel Doc EQ imaging system (Bio-Rad). PCR products were sequenced at Sangon Biotech. The resultant sequences were entered into DNAStar software (DNASTAR Inc., Madison, WI, United States), and sequence alignments were performed by the ClustalW method.

### Evaluation of the RAA Assay Using Clinical Samples

To evaluate the performance of the RAA assay directly on clinical samples, 112 clinical stool samples were tested using the established RAA methods in this study for identifying *bla*_NDM_ genes. All the RAA test results were compared with those from the standard PCR assay.

### Phenotypes of the NDM-Positive Clinical Isolates

Antimicrobial susceptibility testing of the *bla*_NDM_-positive isolates was initially performed using the VITEK 2 compact system. The MIC values for amikacin, tobramycin, ciprofloxacin, levofloxacin, tigecycline, doxycycline, minocycline, co-trimoxazole, aztreonam, cefepime, ceftazidime, ceftriaxone, cefuroxime, cefoperazone/sulbactam, ticarcillin/clavulanic acid, piperacillin/tazobactam, imipenem, and meropenem were determined by VITEK 2 AST-N335 cards. *Escherichia coli* ATCC25922 and *Pseudomonas aeruginosa* ATCC27853 were used for quality control. The concentration gradient-based E-test strip method was used to double check the MIC values for imipenem and meropenem in the *in vitro* susceptibility tests. The 2020 Clinical Laboratory Standards Institute’s threshold was used to as reference.

### Statistical Analysis

The trials were performed in triplicate. The kappa and *p*-values of the RAA and standard PCR assays (with sequencing) were calculated. The statistical analysis was conducted with SPSS 21.0 (IBM, Armonk, NY, United States).

## Results

### Primer Design for the RAA Assay

**FIGURE 1:**
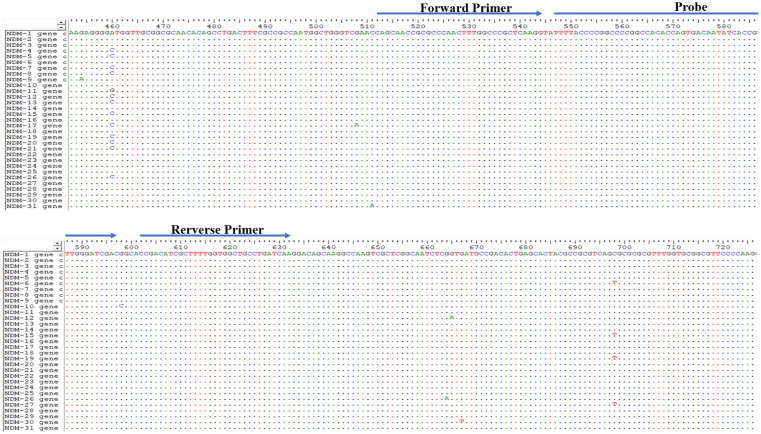
Primer and probe regions in the 31 *bla*_NDM_ variants.

Since the first NDM sequence (*bla*_NDM__–__1_) was released in 2009 (GenBank accession number KU341526.1), 31 variants have been identified. The genome sequences of the *bla*_NDM_ genes are almost identical. The primers and probe were manually designed to bind within the conserved regions according to the principles of RAA primer and probe design (Figure 1 and Table 2).

**TABLE 2:** Primers used to amplify the *bla*_NDM_ genes.

**Primer**	**Sequence**	**Position**
NDM-F	CAGCAACCGCGCCCAACTTTG GCCCGCTCAAGG	512-544^a^
NDM-R	TTGATCAGGCAGCCACCAAAA GCGATGTCGG	602-632^a^
NDM-P	TTTTACCCCGGCCCCGGCC ACACCAGTGACAA[FAM-dt][THF][BHQ-dt]CACCGTTGGGATCGAC[3′-block]	547-597^a^
16S-F	TGGAGCATGTGGTTTAATTC GATGCAACGC	1022285-1022314^b^
16S-R	GGATAAGGGTTGCGCTCGTT GCGGGACTTAA	1022432-1022462^b^
16S-P	TGACATCCACAGAACTTTCCAGA GATGGATTGG[FAM-dT]G[THF]C [BHQ-dT] TCGGGAACTGTGAGAC [3′-block]	1022334-1022387^b^

*^a^GenBank accession number KU341526.1.*

*^b^GenBank accession number NZ_CP006659.2.*

*FAM, 6-carboxyfluorescein; THF, tetrahydrofuran; BHQ, black hole quencher; C3-spacer, 3′-phosphate blocker.*

### Analytical Specificity of the RAA Assay

**FIGURE 2:**
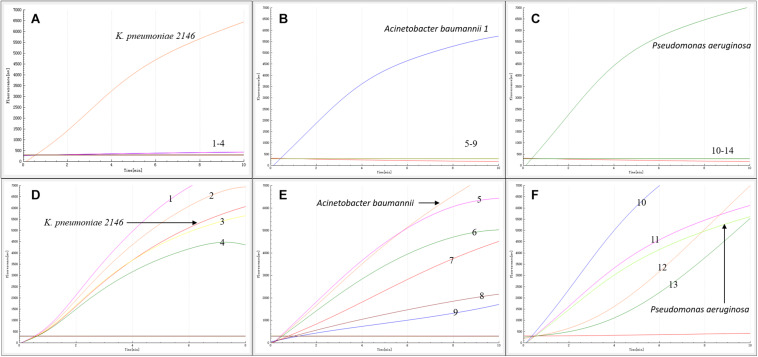
Specificity of the RAA assay. *Klebsiella pneumoniae* 2146, *Acinetobacter baumannii* 1 and *Pseudomonas aeruginosa* produced amplification signals, while the other *bla*_NDM_-lacking bacterial types were negative **(A–C)**. All the bacterial types produced 16S rRNA gene amplification signals **(D–F)**. 1: *K. oxytoca*, 2: *A. baumannii 2*, 3: *K. pneumoniae* 700603, 4: *P. aeruginosa* ATCC27853, 5: *S. sonnei*, 6: *E. coli* 25922, 7: *C. jejuni*, 8: *E. aerogenes*, 9: *P. mirabilis*, 10: *S. enteritidis*, 11: *E. cloacae*, 12: *C. freundii*, 13: *P. mirabilis.*

As shown in Figures 2A–C, in contrast with the other bacterial samples and water control, only *K. pneumoniae* 2146-, *A. baumannii*-, and *P. aeruginosa* DNA-containing samples produced amplification signals. However, all the bacterial types produced amplification signals from the 16S rRNA gene (Figures 2D–F). Hence, the RAA assay for the detection of *bla*_NDM_ was specific (100%).

### Analytical Sensitivity of the RAA Assay

**FIGURE 3:**
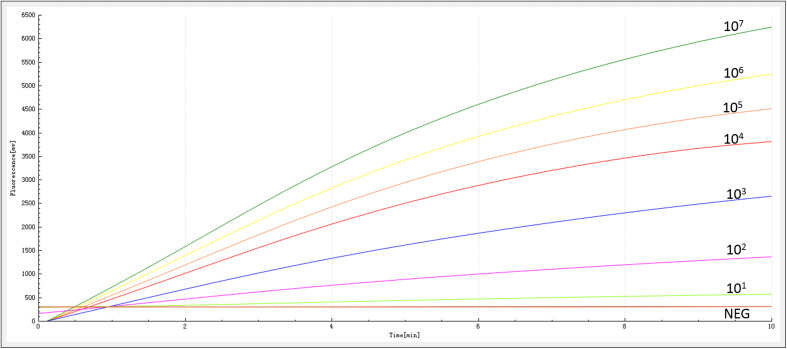
Sensitivity of the RAA assay. An increase in the fluorescence signal was observed from 1 × 10^7^ to 1 × 10^1^ copies/reaction.

The sensitivity of the RAA assay for *bla*_NDM_ detection was determined using a panel of serially diluted recombinant plasmids and bacterial genomic DNA containing the *bla*_NDM_ gene. As shown in Figure 3, an increase in the fluorescence signal was observed from 1 × 10^7^ to 1 × 10^1^ copies/reaction. The detection limit of the RAA assay was 20 copies per reaction.

### Evaluating the RAA Assay on Clinical Samples

**FIGURE 4:**
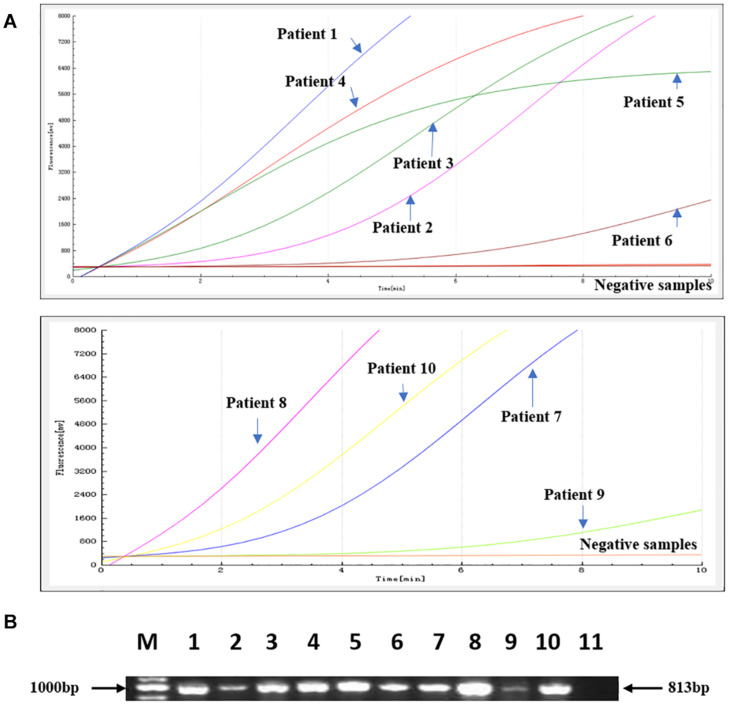
Comparison of the RAA detection results with standard PCR on 112 clinical samples. Ten positive samples were identified. **(A)** The RAA detection results from the 10 positive samples. **(B)** The PCR results from the same 10 positive samples. M: Marker, 1–10: The 10 positive samples for patients 1–10; 11: negative control.

The RAA assay was then evaluated with 112 stool samples, and the results were verified by standard PCR. From these 112 clinical samples, 10 were positive for *bla*_NDM_. All the results were 100% consistent with the results from the standard PCR assay. No significant differences between the detection results from RAA and PCR were observed (Figure 4).

### Culture and Isolation of the *bla*_NDM_-Positive Clinical Samples

Fifteen carbapenem-resistant bacterial types were isolated from the 10 aforementioned samples including *E. coli*, *K. pneumoniae*, *A. baumannii*, *C. freundii*, *K. oxytoca*, *A. junii*, and *P. mirabilis*. Interestingly, more than one bacterial type was identified in three samples, and *bla*_NDM__–__1_ and *bla*_NDM__–__5_ existed in one patient’s sample. After comparing the sequences of these bla_NDM_ genes in GenBank, 11 *bla*_NDM__–__1_ and 4 *bla*_NDM__–__5_ types were confirmed (Table 3).

**TABLE 3:** RAA detection results for clinical samples.

**Sample ID**	**RAA**	**PCR**	**Culture**	**Sequencing**
EYS1-1	+	+	*E. coli*	NDM-1
ESY1-2	+	+	*E. coli*	NDM-5,
ESY1-3	+	+	*K. pneumoniae*	NDM-1
ESY2-1	+	+	*K. pneumoniae*	NDM-1
ESY2-2	+	+	*K. oxytoca*	NDM-1
ESY2-3	+	+	*C. freundii*	NDM-1
ESY3-1	+	+	*C. freund*	NDM-1
ESY3-2	+	+	*C. freund*	NDM-5
ESY4	+	+	*A. junii*	NDM-1
ESY5	+	+	*K. pneumoniae*	NDM-1
ESY6	+	+	*K. pneumoniae*	NDM-5
ESY7	+	+	*E. coli*	NDM-5
ESY8	+	+	*A. baumannii*	NDM-1
ESY9	+	+	*A. baumannii*	NDM-1
ESY10	+	+	*P. mirabilis*	NDM-1

### Clinical Information on *bla*_NDM_-Positive Isolates From Patients

To further understand the characteristics of *bla*_NDM_, the clinical characteristics of the three patients (ESY1, ESY2, and ESY3) from whom more than one *bla*_NDM__–_positive isolates and *bla*_NDM_ variants were identified were investigated (Supplementary Table 1). The diagnoses of these patients included chylous ascites and leukemia. All of these three patients had long histories of antibiotic therapy. Their clinical characteristics and antibiotic treatments were consistent with the culture and RAA detection results.

### Susceptibility Test Results for the *bla*_NDM_-Positive Bacteria

Following testing with the VITEK 2 compact system, most of the carbapenem-resistant *Enterobacterales* (CRE) types were resistant to cephalosporins, cefoperazone-sulbactam, piperacillin-tazobactam, ticarcillin-clavulanic acid, aztreonam, co-trimoxazole, and carbapenems. Overall, five, nine, and six bacteria types, respectively, were resistant to tobramycin, ciprofloxacin, and levofloxacin. Seven of the bacterial types were susceptible to doxycycline and minocycline. Amikacin and tigecycline showed excellent antibacterial activity against the CRE types (Supplementary Table 2).

## Discussion

In recent years, with the widespread use of carbapenems, CRE have become prevalent in the clinic (van Loon et al., 2017; Potter et al., 2016). The successful clinical treatment and control of CRE-related infections has faced great challenges.

*bla*_NDM_ is an MBL carbapenemase type usually seen in Asia (Safavi et al., 2020). The prevalence of NDM-producing *E. coli* was reported to be 82.6, 12.9, 1.5, 1.0, and 2.0% in Asia, Europe, America, Africa, and Oceania, respectively (Dadashi et al., 2019). The Chinese CRE Network showed that the *bla*_DM_ production rate for carbapenemase-producing clinical isolates was 33.5% and these isolates were widely distributed in *K. pneumoniae*, *E. coli*, *E. cloacae*, and other species (Zhang et al., 2018). Dissemination of *bla*_NDM_ through horizontal gene transfer is a potential threat to society. The detection rate for *bla*_NDM_ in children was found to be comparably higher than in adults, with rates of 49% for CRE strains in children and 20.6% in adults (Fan et al., 2019). Development of a sensitive and reliable test to identify *bla*_NDM_ directly in pediatric samples is therefore a priority for early diagnosis and infection control.

In this study, we developed an RAA assay to detect *bla*_NDM_ directly in clinical specimens. This assay has proved to be highly specific and sensitive, as shown by its successful use in identifying SARS-CoV-2, hepatitis B virus, adenovirus, respiratory syncytial virus, salmonella, and other pathogens (Zhang et al., 2017; Fan et al., 2019; Qi et al., 2019; Shen et al., 2019; Wang et al., 2019; Xue et al., 2020a,b). In our tests, its sensitivity was 20 copies per reaction (0.1 pg/μL), which is comparable with the detection levels of other rapid detection methods, such as loop-mediated isothermal amplification (2.6–25.8 copies/reaction, 10.7 pg/μL) (Naas et al., 2011; Liu et al., 2012; Vasoo et al., 2013; Rathinasabapathi et al., 2015; Lund et al., 2018; Moreira et al., 2018; Bordin et al., 2019), and multiplex real-time PCR (7.5–1,000 cfu/ml). The primers we designed targeted the conserved region of the *bla*_NDM_ gene among 28 variants, thereby avoiding any potential cross amplification with other genes. The results confirm that our RAA assay is highly specific by only providing a positive result for the *bla*_NDM_ gene, and no cross-reactions in other bacterial types without *bla*_NDM_ genes.

Even though multiplex real-time PCR and LAMP are also highly sensitive and specific, their whole reaction times are relatively long at approximately 1–2 h. The RAA detection method takes the shortest time, producing its results within 20 min, which makes it superior to these other methods. In addition, RAA is cheaper than other isothermal amplification techniques, such as LAMP and nucleic acid sequence-based amplification (NASBA).

To investigate the use of this method in clinical samples, we tested 112 clinical pediatric samples. Ten of them were *bla*_NDM_-positive, and the RAA assay showed 100% percent agreement with the PCR method. These results confirm the validity of the RAA method for the rapid detection of *bla*_NDM_ in clinical samples.

We further investigated the characteristics of *bla*_NDM_-positive carbapenem-resistant bacteria in children using the 15 bacteria types we identified (which included 4 *K. pneumoniae*, 3 *E. coli*, 3 *C. freundii*, 2 *A. baumannii*, 1 *A. junii*, 1 *K. oxytoca*, and 1 *P. mirabilis*) in the 10 samples. Our findings are consistent with those from other studies showing that *K. pneumoniae*, *E. coli*, and *C. freundii* were the most prevalent types in CRE strains (Fan et al., 2019). Here, the sequencing results showed that four of the bacteria types were positive for *bla*_NDM__–__1_ and two for *bla*_NDM__–__5_, which was consistence with the other studies showing that *bla*_NDM__–__1_ and *bla*_NDM__–__5_ are disseminated among *Enterobacterales* in children in China (Fan et al., 2019; Li et al., 2020; Tian et al., 2020).

Interestingly, we isolated more than one carbapenem-resistant type from three patients and found that bla_NDM__–__1_ and bla_NDM__–__5_ coexisted in one patient. The clinical characteristics of the three patients showed that two were patients from the hematology department and one was a patient in the intensive care unit where multiple antibiotics are often used. We then analyzed the antibiotic resistance characteristics of the 15 isolates. The MIC results showed these isolates were highly resistant to cephalosporins, cefoperazone-sulbactam, piperacillin-tazobactam, ticarcillin-clavulanic acid, aztreonam, co-trimoxazole, and carbapenems but susceptible to tigecycline and amikacin. The histories of antibiotic use in these patients were consistent with the detection results.

In summary, the RAA assay has high specificity and sensitivity in detecting *bla*_NDM_ genes in clinical samples. By providing a simple, rapid, and reliable method for *bla*_NDM_ detection, this assay may prove a great help for clinical treatment and antibiotic use. The common existence of *bla*_NDM_ and multi-drug resistance remains a big challenge for the clinical treatment of infections.

## Data Availability Statement

The original contributions presented in the study are included in the article/Supplementary Material, further inquiries can be directed to the corresponding author/s.

## Ethics Statement

Written informed consent was obtained from the minor(s)’ legal guardian/next of kin for the publication of any potentially identifiable images or data included in this article.

## Author Contributions

JY, YW, and DQ designed the study. YF, GX, CY, JC, HZ, RZ, LG, and WX performed the experiments. NL, SL, SD, WZ, HY, JT, LM, and TZ analyzed the results. YF and GX wrote the manuscript. JY, YW, and DQ revised the manuscript. All authors read and approved the final manuscript.

## Conflict of Interest

The authors declare that the research was conducted in the absence of any commercial or financial relationships that could be construed as a potential conflict of interest.

## Footnotes

1 http://www.ncbi.nlm.nih.gov/

2 http://www.oligoevaluator.com

## References

[B1] Bordin A. Trembizki E. Windsor M. Wee R. Tan L. Y. Buckley C. (2019). Evaluation of the SpeeDx Carba (beta) multiplex real-time PCR assay for detection of NDM, KPC, OXA-48-like, IMP-4-like and VIM carbapenemase genes. *BMC Infect. Dis.* 19:571. 10.1186/s12879-019-4176-z 31266450 PMC6604329

[B2] Dadashi M. Yaslianifard S. Hajikhani B. Kabir K. Owlia P. Goudarzi M. (2019). Frequency distribution, genotypes and prevalent sequence types of New Delhi metallo-β-lactamase-producing *Escherichia coli* among clinical isolates around the world: a review. *J. Glob. Antimicrob. Resist.* 19 284–293. 10.1016/j.jgar.2019.06.008 31212107

[B3] Fan G. H. Shen X. X. Li F. Li X. N. Bai X. D. Zhang R. Q. (2019). Development of an Internally Controlled Reverse Transcription Recombinase-aided Amplification Assay for the Rapid and Visual Detection of West Nile Virus. *Biomed. Environ. Sci.* 32 926–929. 10.3967/bes2019.116 31918798

[B4] Farhat N. Khan A. U. (2020). Evolving trends of New Delhi Metallo-betalactamse (NDM) variants: a threat to antimicrobial resistance. *Infect. Genet. Evol.* 2020:104588. 10.1128/AAC.00774-09 33038522

[B5] Hong D. J. Bae I. K. Jang I. H. Jeong S. H. Kang H. K. Lee K. (2015). Epidemiology and characteristics of metallo-β-lactamase-producing *Pseudomonas aeruginosa*. *Infect. Chemother.* 47 81–97. 10.3947/ic.2015.47.2.81 26157586 PMC4495280

[B6] Khan A. U. Maryam L. Zarrilli R. (2017). Structure, genetics and worldwide spread of New Delhi Metallo-β-lactamase (NDM): a threat to public health. *BMC Microbiol.* 17:101. 10.1186/s12866-017-1012-8 28449650 PMC5408368

[B7] Kumarasamy K. K. Toleman M. A. Walsh T. R. Bagaria J. Butt F. Balakrishnan R. (2010). Emergence of a new antibiotic resistance mechanism in India, Pakistan, and the UK: a molecular, biological, and epidemiological study. *Lancet Infect. Dis.* 10 597–602. 10.1016/S1473-3099(10)70143-2 20705517 PMC2933358

[B8] Li J. Yu T. Tao X. Y. Hu Y. M. Wang H. C. Liu J. L. (2020). Emergence of an NDM-5-producing *Escherichia coli* sequence type 410 clone in infants in a Children’s hospital in China. *Infect. Drug Resist.* 13 703–710. 10.2147/IDR.S244874 32184632 PMC7054006

[B9] Liu W. Zou D. Li Y. Wang X. He X. Wei X. (2012). Sensitive and rapid detection of the new Delhi metallo-beta-lactamase gene by loop-mediated isothermal amplification. *J. Clin. Microbiol.* 50 1580–1585. 10.1128/JCM.06647-11 22357496 PMC3347096

[B10] Lund M. Petersen M. B. Jørgensen A. L. Paulmann D. Wang M. (2018). Rapid real-time PCR for the detection of IMP, NDM, VIM, KPC and OXA-48 carbapenemase genes in isolates and spiked stool samples. *Diagn. Microbiol. Infect. Dis.* 92 8–12. 10.1016/j.diagmicrobio.2018.04.002 29776709

[B11] Lyon J. A. (1985). Imipenem/cilastatin: the first carbapenem antibiotic. *Drug Intellig. Clin. Pharm.* 19 895–899. 3910385

[B12] Moreira M. G. Barreto L. M. Dos Santos V. L. Monteiro A. S. Nobre V. Dos Santos S. G. (2018). Rapid detection of the New Delhi metallo-b-lactamase 1 (NDM-1) gene by loop-mediated isothermal amplification (LAMP). *J. Clin. Lab. Anal.* 32:e22323. 10.1002/jcla.22323 28960568 PMC6816916

[B13] Naas T. Ergani A. Carrër A. Nordmann P. (2011). Real-time PCR for detection of NDM-1 carbapenemase genes from spiked stool samples. *Antimicrob. Agents Chemother.* 55 4038–4043. 10.1128/AAC.01734-10 21690281 PMC3165338

[B14] Potter R. F. D’Souza A. W. Dantas G. (2016). The rapid spread of carbapenem-resistant *Enterobacteriaceae*. *Drug Resist. Update* 29 30–46. 10.1016/j.drup.2016.09.002 27912842 PMC5140036

[B15] Qi J. Li X. Zhang Y. Shen X. Song G. Pan J. (2019). Development of a duplex reverse transcription recombinase-aided amplification assay for respiratory syncytial virus incorporating an internal control. *Arch. Virol.* 164 1843–1850. 10.1007/s00705-019-04230-z 31053978 PMC7086889

[B16] Rahman M. Prasad K. N. Gupta S. Singh S. Singh A. Pathak A. (2018). Prevalence and molecular characterization of New Delhi metallo-beta-lactamases in multidrug-resistant *Pseudomonas aeruginosa* and *Acinetobacter baumannii* from India. *Microb. Drug Resist.* 24 792–798. 10.1089/mdr.2017.0078 29058515

[B17] Rathinasabapathi P. Hiremath D. S. Arunraj R. Parani M. (2015). Molecular detection of New Delhi metallo-beta-lactamase-1 (NDM-1) positive bacteria from environmental and drinking water samples by loop mediated isothermal amplification of bla NDM-1. *Indian J. Microbiol.* 55 400–405.27. 10.1007/s12088-015-0540-x 26543265 PMC4627955

[B18] Safavi M. Bostanshirin N. Hajikhani B. Yaslianifard S. van Belkum A. Goudarzi M. (2020). Global genotype distribution of human clinical isolates of New Delhi metallo-β-lactamase-producing *Klebsiella pneumoniae*; a systematic review. *J. Glob. Antimicrob. Resist.* 23 420–429. 10.1016/j.jgar.2020.10.016 33157280

[B19] Shen X. X. Qiu F. Z. Shen L. P. Yan T. F. Zhao M. C. Qi J. J. (2019). A rapid and sensitive recombinase aided amplification assay to detect hepatitis B virus without DNA extraction. *BMC Infect. Dis.* 19:229. 10.1186/s12879-019-3814-9 30836947 PMC6402085

[B20] Tian D. Wang B. Zhang H. Pan F. Wang C. Shi Y. (2020). Dissemination of the blaNDM-5 Gene via IncX3-type plasmid among *Enterobacteriaceae* in Children. *mSphere* 5:e00699-19. 10.1186/s13756-018-0349-6 31915216 PMC6952193

[B21] Tooke C. L. Hinchliffe P. Bragginton E. C. Colenso C. K. Hirvonen V. Takebayashi Y. (2019). β-Lactamases and β-lactamase inhibitors in the 21st Century. *J. Mol. Biol.* 431 3472–3500. 10.1016/j.jmb.2019.04.002 30959050 PMC6723624

[B22] Trecarichi E. M. Tumbarello M. (2017). Therapeutic options for carbapenem-resistant *Enterobacteriaceae* infections. *Virulence* 8 470–484. 10.1080/21505594.2017.1292196 28276996 PMC5477725

[B23] van Loon K. Voor In ’t Holt A. F. Vos M. C. (2017). A systematic review and meta-analyses of the clinical epidemiology of carbapenem-resistant *Enterobacteriaceae*. *Antimicrob. Agents Chemother.* 62:e01730-17. 10.1128/AAC.01730-17 29038269 PMC5740327

[B24] Vasoo S. Cunningham S. A. Kohner P. C. Mandrekar J. N. Lolans K. Hayden M. K. (2013). Rapid and direct real-time detection of blaKPC and blaNDM from surveillance samples. *J. Clin. Microbiol.* 51 3609–3615. 10.1128/JCM.01731-13 23966498 PMC3889741

[B25] Wang R. H. Zhang H. Zhang Y. Li X. N. Shen X. X. Qi J. J. (2019). Development and evaluation of recombinase-aided amplification assays incorporating competitive internal controls for detection of human adenovirus serotypes 3 and 7. *Virol. J.* 16:86. 10.1186/s12985-019-1178-9 31262315 PMC6604330

[B26] Xue G. Li S. Zhang W. Du B. Cui J. Yan C. (2020a). Reverse-transcription recombinase-aided amplification assay for rapid detection of the 2019 novel coronavirus (SARS-CoV-2). *Anal. Chem.* 92 9699–9705. 10.1021/acs.analchem.0c01032 32441935

[B27] Xue G. Li S. Zhao H. Yan C. Feng Y. Cui J. (2020b). Use of a rapid recombinase-aided amplification assay for *Mycoplasma pneumoniae* detection. *BMC Infect. Dis.* 20:79. 10.1186/s12879-019-4750-4 31992210 PMC6988361

[B28] Yang Y. Guo Y. Yin D. Zheng Y. Wu S. Zhu D. (2020). In vitro activity of cefepime-zidebactam, ceftazidime-avibactam, and other comparators against clinical isolates of enterobacterales, *Pseudomonas aeruginosa*, and *Acinetobacter baumannii*: results from China antimicrobial surveillance network (CHINET) in 2018. *Antimicrob. Agents Chemother.* 65:e-1726-20. 10.1128/AAC.01726-20 33139291 PMC7927829

[B29] Yong D. Toleman M. A. Giske C. G. Cho H. S. Sundman K. Lee K. (2009). Characterization of a new metallo-beta-lactamase gene, bla(NDM-1), and a novel erythromycin esterase gene carried on a unique genetic structure in *Klebsiella pneumoniae* sequence type 14 from India. *Antimicrob. Agents Chemother.* 53 5046–5054. 19770275 10.1128/AAC.00774-09 PMC2786356

[B30] Zhang X. Guo L. Ma R. Cong L. Wu Z. Wei Y. (2017). Rapid detection of *Salmonella* with recombinase aided amplification. *J. Microbiol. Methods* 139 202–204. 10.1016/j.mimet.2017.06.011 28619662

[B31] Zhang Y. Wang Q. Yin Y. Chen H. Jin L. Gu B. (2018). Epidemiology of carbapenem-resistant *Enterobacteriaceae* infections: report from the China CRE network. *Antimicrob. Agents Chemother.* 62:e01882-17. 10.1128/AAC.01882-17 29203488 PMC5786810

