# Supplementary material for: Rapid Detection of New Delhi Metallo-β-Lactamase Gene Using Recombinase-Aided Amplification Directly on Clinical Samples From Children
Source: Front Microbiol. 2021 Jul 22;12:691289. doi: 10.3389/fmicb.2021.691289 (PMC8339468; doi:10.3389/fmicb.2021.691289)
Supplement: Supplementary file 1 [file Table_1.DOCX]

Supplemental table 1. Clinical information for the three *bla*_NDM_-positive patients

|  | Patient 1 | Patient 2 | Patient 3 |
| --- | --- | --- | --- |
| Age | 1m16d | 11y20d | 8m29d |
| Gender | Male | Male | Female |
| Diagnosis | Chylous ascites | leukemia | leukemia |
| Length of stay | 26d | 22d | 39d |
| Antibiotic | Imipenem  Latamoxef | Imipenem  Cefepime | Imipenem Linezolid  Cefoperazone Sulbactam |

Y, year; M, month; D, day

Supplemental table 2. Antibiotic susceptibility profiles of the *bla*_NDM_-positive isolates

| **Patient ID** | ESY1 | | | ESY2 | | | ESY3 | | ESY4 | ESY5 | ESY6 | ESY7 | ESY8 | ESY9 | ESY10 |
| --- | --- | --- | --- | --- | --- | --- | --- | --- | --- | --- | --- | --- | --- | --- | --- |
| **Isolates** | *Eco*  *bla_ndm-1_* | *Eco*  *bla_ndm-5_* | *Kpn*  *bla_ndm-1_* | *Kpn*  *bla_ndm-1_* | *Cfr*  *bla_ndm-1_* | *Kox*  *bla_ndm-1_* | *Cfr*  *bla_ndm-1_* | *Cfr*  *bla_ndm-5_* | *Aju*  *bla_ndm-1_* | *Kpn*  *bla_ndm-5_* | *Kpn*  *bla_ndm-1_* | *Eco*  *bla_ndm-5_* | *Aba*  *bla_ndm-1_* | *Aba*  *bla_ndm-1_* | *Pmi*  *bla_ndm-1_* |
| AK | ≤2 | ≤2 | ≤2 | ≤2 | ≤2 | ≤2 | ≤2 | ≤2 | ≤2 | ≤2 | ≥64 | ≤2 | ≤2 | ≥16 | ≤2 |
| TOB | 8 | 8 | 8 | ≤1 | ≥16 | ≤1 | ≤1 | ≥16 | ≥16 | 8 | ≥16 | ≤1 | ≤1 | ≥16 | ≤1 |
| CIP | 1(R) | 1 | 1 | ≤0.25 | 2 | ≤0.25 | ≤0.25 | 2 | ≤0.25 | ≥4 | ≥4 | ≥4 | 2 | ≥4 | ≤0.25 |
| LEV | 1(I) | 1 | 1 | 0.5 | 2 | 0.5 | 0.5 | 2 | ≤0.12 | 2 | ≥8 | ≥8 | 1 | 4 | ≤0.25 |
| SMZ | ≥320 | ≥320 | ≤20 | ≤20 | ≥320 | ≤20 | ≤20 | ≥320 | ≥320 | ≥320 | ≤20 | ≥320 | ≥320 | ≥320 | ≥320 |
| TGC | ≤0.5 | ≤0.5 | 1 | 2 | ≤0.5 | 2 | 2 | ≤0.5 | ≤0.5 | ≤0.5 | 2 | 1 | ≤0.5 | ≤0.5 | ≤0.5 |
| DO | 8 | 8 | 1 | 8(I) | ≥16 | 4 | 4 | ≥16 | ≤0.5 | ≥16 | ≥16 | 4 | 4 | 4 | 4 |
| MH | 4 | 2 | 2 | ≥16 | ≥16 | ≥16 | ≥16 | ≥16 | ≤1 | ≥16 | ≥16 | 4 | 4 | 4 | 4 |
| ATM | ≤1 | ≤1 | 32 | ≥64 | ≤1 | ≥64 | ≥64 | ≤1 | 1 | ≥64 | ≥64 | ≥64 | 4 | ≥64 | ≤1 |
| CAZ | ≥64 | ≥64 | ≥64 | ≥64 | ≥64 | ≥64 | ≥64 | ≥64 | 32 | ≥64 | ≥64 | ≥64 | ≥64 | ≥64 | ≥64 |
| CRO | ≥64 | ≥64 | ≥64 | ≥64 | ≥64 | ≥64 | ≥64 | ≥64 | ≥64 | ≥64 | -- | ≥64 | ≥64 | ≥64 | 8 |
| FEP | 16 | ≥32 | 8 | ≥32 | ≥32 | ≥32 | ≥32 | ≥32 | 1 | ≥64 | ≥32 | ≥64 | ≥64 | ≥64 | 4 |
| CXM | ≥64 | ≥64 | ≥64 | ≥64 | ≥64 | ≥64 | ≥64 | ≥64 | ≥64 | ≥64 | -- | ≥64 | ≥64 | ≥64 | ≥64 |
| CZ | ≥64 | ≥64 | ≥64 | ≥64 | ≥64 | ≥64 | ≥64 | ≥64 | ≥64 | ≥64 | ≥64 | ≥64 | ≥64 | ≥64 | ≥64 |
| CFP | ≥64 | ≥64 | ≥64 | ≥64 | ≥64 | ≥64 | ≥64 | ≥64 | 8 | 16 | ≥64 | 8 | 32 | 32 | 16 |
| TIM | ≥128 | ≥128 | ≥128 | ≥128 | ≥128 | ≥128 | ≥128 | ≥128 | 8 | ≥128 | ≥128 | ≥128 | ≥128 | ≥128 | 16 |
| TIP | ≥128 | ≥128 | ≥32 | ≥128 | ≥128 | ≥128 | ≥128 | ≥128 | ≥128 | ≥128 | ≥128 | ≥128 | ≥128 | ≥128 | 16 |
| IMP | ≥16 | ≥16 | ≥16 | ≥16 | ≥16 | ≥16 | ≥16 | ≥16 | 4 | ≥16 | ≥16 | ≥16 | ≥16 | ≥16 | 4 |
| MEM | ≥16 | ≥16 | ≥16 | ≥16 | ≥16 | ≥16 | ≥16 | ≥16 | 4 | 8 | ≥16 | ≥16 | ≥16 | ≥16 | 4 |
| E-test for IMP | 16 | 16 | 16 | 32 | 16 | 16 | 16 | 16 | 4 | 8 | 32 | 16 | 16 | 16 | 8 |
| E-test for MEM | 16 | 16 | 16 | 32 | 16 | 16 | 16 | 16 | 4 | 8 | 32 | 16 | 16 | 16 | 8 |

AZK: aztreonam, TOB: tobramycin, CIP: ciprofloxacin, LEV: levofloxacin, SMZ: Co-trimoxazole, TGC: tigecycline, DO: doxycycline, MH: minocycline, ATM: aztreonam, CAZ: ceftazidime, CRO: ceftriaxone, FEP: cefepime, CXM: cefuroxime, CZ: cefazolin, CFP: cefoperazone/sulbactam, TIM: ticarcillin/clavulanic acid, TIP: piperacillin/tazobactam, IMP: imipenem, MEM: meropenem.

*Eco: Escherichia coli, Kpn: Klebsiella pneumoniae, Cfr: Citrobacter freundii, Kox: Klebsiella oxytoca, Aju: Acinetobacter junii, Aba: Acinetobacter baumannii, Pmi: Proteus mirabilis*
